# Exploring the Advantages and Techniques of Intraoperative Transcholecystic Methylene Blue Injection in Laparoscopic Cholecystectomy

**DOI:** 10.7759/cureus.64205

**Published:** 2024-07-10

**Authors:** Pawan K Jha, Pradeep Jaiswal, Shitiz Gera, Rinku Kumari, Kaushalendra Kumar, Rohit Darjee, Deepak Pankaj

**Affiliations:** 1 General Surgery, Indira Gandhi Institute of Medical Sciences, Patna, IND

**Keywords:** methylene blue, laparoscopic cholecystectomy, intraoperative, gallbladder, bile duct

## Abstract

Introduction

With the use of advanced instruments and techniques, the reported incidence of bile duct injury is low; however, the actual frequency might be slightly higher than reported. Most surgeons might encounter bile duct injury or bile duct-related complications in their early training days. Nevertheless, with newer techniques and technologies, cases of bile duct injuries have been mostly observed in open cholecystectomy. The predominant cause of injury is the misinterpretation of the anatomy of the bile duct, cystic duct, or aberrant right sectoral hepatic duct. Laparoscopic cholecystectomy is currently the gold standard of therapy for cholecystitis.

Materials and methods

The study was conducted in the Department of General Surgery at the Indira Gandhi Institute of Medical Sciences in Patna, after obtaining clearance from the ethics committee. The duration of the study was one year.

Results

A total of 50 patients were enrolled in the study, whose ages ranged from 20 to 55 years. They were predominantly female. The mean operative time was 68.5 ± 8.7 minutes. There were no cases of conversion to an open procedure, bile duct injury, or biliary stricture.

Conclusion

The injection of methylene blue into the gallbladder fundus during laparoscopic cholecystectomy is a practical, affordable, and simple procedure that does not require any special equipment or radiation exposure for the improved delineation of the gallbladder and biliary system. The use of intraoperative methylene blue could be a low-cost and simple alternative for safe laparoscopic cholecystectomy.

## Introduction

The reported incidence of bile duct injury is around 0.2-0.5% when using all the currently available instruments and techniques [1]. However, the true rate could be slightly higher. In general, half of all surgeons might encounter bile duct injury or bile duct-related complications in their early training days [2]. Nevertheless, with newer techniques and technologies, cases of bile duct injuries have been mostly observed in open cholecystectomy [3].

The predominant cause of injury by a surgeon is the misinterpretation of the anatomy of the bile duct, cystic duct, or aberrant right sectoral hepatic duct [4,5].

Laparoscopic cholecystectomy is currently the gold standard of therapy for cholecystitis [6]. In comparison with open cholecystectomy, cholecystectomy via laparoscopy has various benefits, which include less postoperative pain, better cosmesis, a shorter hospital stay, and an early return to work [7].

Certain factors make it challenging to perform laparoscopic cholecystectomy, including chronic cholecystitis, contracted gallbladder, choledocholithiasis, thick-walled gallbladder (>3 mm), old age (>40 years), American Society of Anesthesiologists (ASA) grade 3 and higher, male sex, obesity, number of previous acute attacks, previous surgery, common bile duct dilation (>6 mm), and preoperative endoscopic retrograde cholangiopancreatography (ERCP) [8-10].

These factors increase the risk of bile duct injuries, conversion to open procedures, prolonged operative time, and longer hospital stays. Injuries to the common bile duct are one of the most unpleasant sequelae experienced by both patients and practitioners. These injuries can lead to severe morbidity and considerable discomfort [11]. Procedures such as intraoperative cholangiography and laparoscopic ultrasound have been used to decrease complications and the time of laparoscopic cholecystectomy. The use of indocyanine green dye during the procedure is an emerging treatment modality; however, it requires costly equipment [12].

The injection of methylene blue offers a readily accessible alternative, distinguished by its ease of learning and implementation, along with minimal setup costs, making it an efficient and economical choice for various medical applications. Following sensitivity testing with methylene blue, it is injected into the gallbladder to better delineate the biliary tract. The present study aimed to examine the feasibility of intraoperative transcholecystic methylene blue injection during laparoscopic cholecystectomy and its effectiveness in delineating the biliary tree.

## Materials and methods

After obtaining clearance from the institutional ethical committee (1153/IEC/IGIMS/2023), this study was conducted in the Department of General Surgery at the Indira Gandhi Institute of Medical Sciences in Patna. Every patient enrolled in the study provided written informed consent. This was a prospective, hospital-based, non-randomized study, and a proforma with patient details was used.

The study was conducted over a period of one year, from June 2023 to May 2024, enrolling a total of 50 patients. All patients had cholecystitis diagnosed according to the Tokyo 18 guidelines [13]. The patients were followed up at two weeks, one month, and six months after surgery.

Patients with previous adverse effects due to methylene blue, known G6PD deficiency, renal or cardiovascular diseases, immunocompromised status, unwillingness to undergo follow-ups, or inability to provide adequate information were excluded from the study.

Preoperative examination involved routine blood tests including complete blood count, liver and kidney function tests, chest X-ray, electrocardiography, and echocardiography in certain cases with potential cardiac complications. In addition, blood pressure, heart rate, and blood sugar levels were assessed.

The patients were informed of the possibility of passage of methylene blue in the urine and the expected intraoperative time, along with potential preoperative, operative, and postoperative complications that might arise due to surgery or anesthesia.

Surgical procedure 

After sensitivity testing with methylene blue on the patient's right forearm and ruling out any hypersensitivity reaction, the procedure was performed under general anesthesia using the four-port American technique. The patient was placed in a reverse Trendelenburg position, slightly elevated on the right side. Proper positioning and draping were applied. The pneumoperitoneum was created using either a Veress needle or an open technique, and two 10 mm ports and two 5 mm ports were created at the usual positions. 

After removing adhesions from the gallbladder, the fundus of the gallbladder was pulled towards the anterior abdominal wall, and all bile was aspirated. An equal amount of 50% diluted methylene blue (approximately 15 to 20 mg) was injected into the gallbladder [11]. To prevent leakage of the methylene blue, the fundus was grasped tightly at the position of injection, or a clip was placed at the site of needle insertion [11]. The dissection of Calot’s triangle was carried out, and the staining of the gallbladder by methylene blue was observed. After some time, the staining of the cystic duct and bile duct was also observed.

When the critical view of safety was achieved, the gallbladder was removed using a hook or a harmonic scalpel with an endo bag. In cases of dye spillage, normal saline was used to irrigate the abdominal cavity. The abdominal cavity was irrigated and suctioned to remove any leaked methylene blue, and a drain was left in situ in the subhepatic space. All ports were closed.

Data analysis 

Data were collected in a pre-made proforma using a Microsoft Excel sheet (Microsoft Corporation, Redmond, Washington), and IBM SPSS Statistics for Windows, Version 20 (Released 2011; IBM Corp., Armonk, New York), was used to analyze the data. The percentage of each variable was analyzed, and variables with the highest and lowest percentages were identified. Continuous and categorical variables were displayed as counts or frequencies. For analysis, these data were effectively arranged in contingency tables. Statistical significance of the association was determined by Fisher’s exact test at a p-value of less than 0.05.

## Results

**Figure 1:**
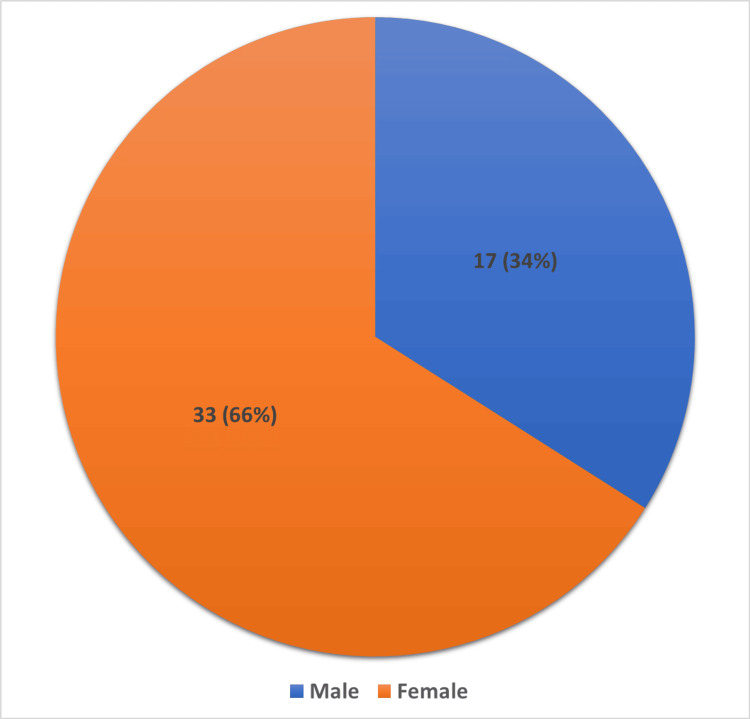
Number (percentage) of males and females in the study

A total of 50 patients were included in the present study. Patients with cholecystitis ranged in age from 20 to 55 years. Most patients were in the age range of 30-40 years (44%), consisting of 13 females and 9 males. On the other hand, the age group of 50-55 years (2%) comprised only one female (Table 1). The mean age of the patients included in the study was 35 ± 2.5 years. A total of 33 patients (66%) were females, and 17 patients (34%) were males (Figure 1). The female-to-male ratio was 1.9:1. Among patients who were included in the study and underwent surgery, the lowest age was 21 years, and the highest age was 53 years.

**Table 1:** Different age groups of patients

Age distribution (years)	Male	Female	Percentage (%)
20-30	6	10	32
30-40	9	13	44
40-50	2	9	22
50-55	0	1	2

**Figure 2:**
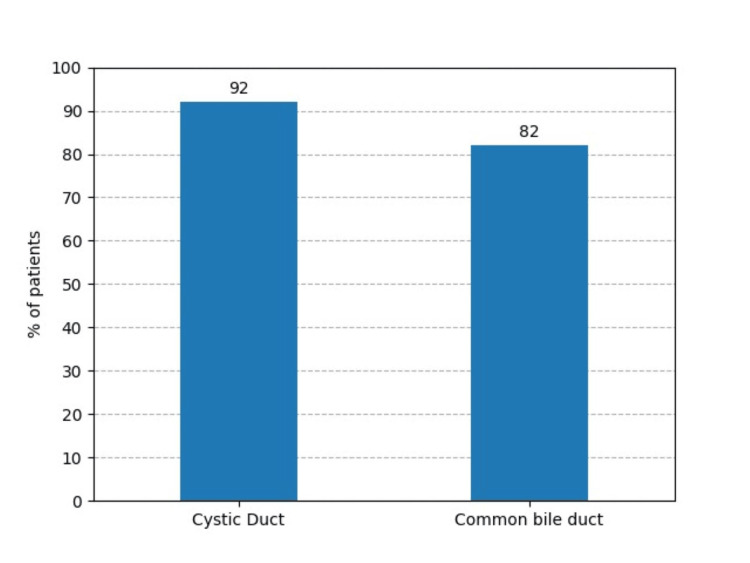
Percentage of patients with staining of the cystic duct (92%) and common bile duct (82%)

**Figure 3:**
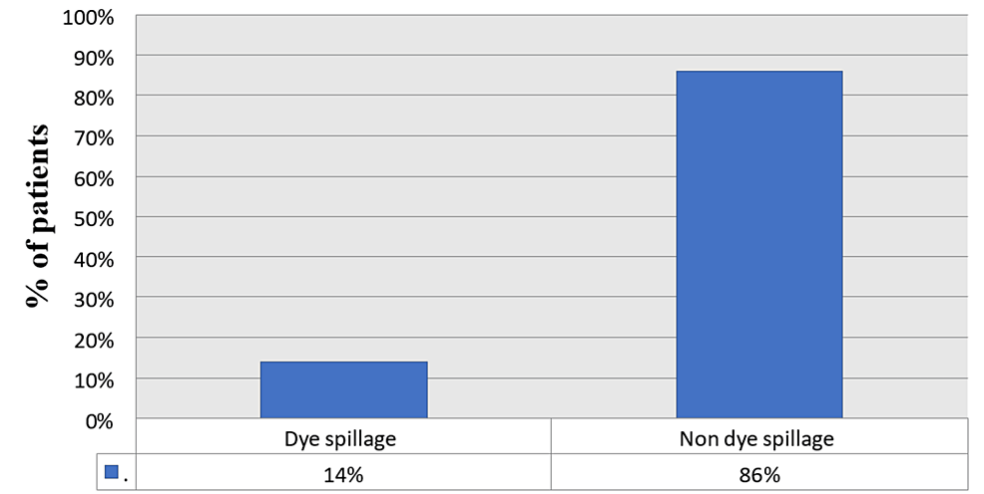
Percentage of patients with dye spillage and without dye spillage

The mean operative time was 68.5 ± 8.7 minutes; the maximum time was 102 minutes, and the minimum time was 35 minutes (Table 2). In 46 cases (92%), the cystic duct was stained, and in 41 cases (82%), the common bile duct and gallbladder were stained with methylene blue (Figure 2). Dye spillage during the procedure was observed in 14% of cases, whereas no spillage of the dye was observed in 86% of cases (Figure 3).

**Table 2:** Operative time of the procedure

Operative time	Duration
Maximum	102 min
Minimum	35 min
Mean ± SD	68.5 ± 8.7 min

Postoperative complications included urine discoloration in five cases (10%) due to infusion of dye, dye extravasation in seven cases (14%) at the point of infiltration in the gallbladder fundus or body, and stone spillage in one case (2%) (Table 3). None of the cases involved biliary leak or biliary stricture, and no case was converted to an open procedure.

**Table 3:** Percentage of patients with postoperative complications and complications related to dye infusion

Complication	Number of patients	Percentage of patients (%)
Conversion to open	None	-
Biliary leak	None	-
Biliary stricture	None	-
Stone spillage	1	2
Dye extravasation	7	14
Urine discoloration	5	10

The cystic duct was not stained with methylene blue dye in four cases (8%), which was observed in patients with a thick-walled gallbladder and multiple stones (Table 4).

**Table 4:** Percentage of patients without staining of the cystic duct according to the number of stones and wall thickness

No staining	Stone	Wall	Number	Percentage	P-value (Fisher’s exact test)
Cystic duct	Single	Thick	0	0%	>0.99
		Thin	0	0%	
	Multiple	Thick	4	8%	
		Thin	0	0%	

The common bile duct was not stained in two cases (4%) with a single stone and a thick-walled gallbladder and in two cases (4%) with multiple stones and a thin-walled gallbladder. This outcome was also observed in five cases (10%) with multiple stones and a thick-walled gallbladder (Table 5).

**Table 5:** Percentage of patients without staining of the common bile duct (CBD) according to the number of stones and wall thickness

No staining	Stone	Wall	Number	Percentage	P-value (Fisher’s exact test)
CBD	Single	Thick	2	4%	>0.99
		Thin	0	0%	
	Multiple	Thick	5	10%	
		Thin	2	4%	

## Discussion

Laparoscopic cholecystectomy is currently the standard surgical modality for the treatment of cholecystitis. The top priority during the procedure should be safety, followed by the completion of the procedure.

Inexperienced surgeons should keep in mind that misidentification of the biliary anatomy is the predominant cause of bile duct injury. Along with varied anatomy, adhesions and inflammation also make the identification of the biliary system difficult.

Injuries to the common bile duct are one of the most undesirable outcomes encountered by both patients and healthcare providers, causing significant morbidity and discomfort. It is the third most frequently litigated general surgical complication in the United States, and two surgeries may be necessary in certain circumstances for permanent bile duct repair [14].

Several methods and rules have been developed to better delineate the biliary anatomy; however, most of them rely on either the surgeons' expertise or costly equipment with radiation exposure [15]. For the delineation of the biliary anatomy, the use of methylene blue to stain the gallbladder may be an easy and safe procedure without any radiation exposure, as indicated by Sari et al. [16].

In our study of 50 patients, whose ages ranged from 20 to 55 years, most were female. The mean operative time for the procedure was 68.5 ± 8.7 minutes, which was slightly shorter than in the study by Abdelaziz et al., where the mean operative time was 78 minutes [17].

There were no cases of conversion to open procedure, bile duct injury, or biliary stricture. Although the results may not be directly attributed to the use of methylene blue, its use ensured a much safer and easier dissection of the biliary system as the boundaries of the biliary system were significantly delineated. With this method, 92% of cystic ducts, 82% of common bile ducts, and all gallbladders were stained. The results were similar to those of the study by Abdelaziz et al., where 96% of cystic ducts and 84% of common bile ducts were stained. However, the results differed from those of the study by Radwan et al., where 80% of cystic ducts and 41.7% of common bile ducts were stained [17,18]. The lack of staining of the cystic duct and CBD may be attributed to the presence of multiple stones and the thickness of the gallbladder wall.

The cystic duct was not stained in 8% of patients, and these patients had a thick-walled gallbladder and multiple stones. The CBD was not stained in nine patients, of whom two had a thick-walled gallbladder and single stones, and seven had multiple stones. Similar results were obtained in the study by Rabie et al., where only 26% of CBDs were delineated in patients with multiple stones [19].

In patients with multiple stones, a thick-walled gallbladder could make it difficult to visualize the CBD. In this study, of seven patients with multiple stones, five had a thick wall, and only two had a normal wall. There was dye spillage in 14% of cases in the present study. Methylene blue extravasation occurred in 11% of cases in the study by Sari et al. and in 18% of cases in the study by Abdelaziz et al. [16,17]. In the study by Almaghraby et al., it was noted in nine cases (18%); there was leakage of the dye from the gallbladder into the abdominal cavity during dye injection, needle removal from the gallbladder, or gallbladder removal [20].

Normal saline (0.9%) was used to irrigate the abdominal cavity, and no adverse effects were observed. During methylene blue injection, it could penetrate into small capillaries, leading to a bluish coloration of the urine, which was observed in five cases (10%).

To address the limitations of the current study, a multicenter trial with a larger sample size is essential. This is particularly important given the study's limited sample size and relatively short observation period. In addition, variations in surgical skill and experience among healthcare providers in different regions and healthcare settings could influence the effectiveness and outcome of the technique.

## Conclusions

The injection of methylene blue into the gallbladder fundus during laparoscopic cholecystectomy is a practical, affordable, and simple procedure that does not require any special equipment or radiation exposure for the delineation of the gallbladder and biliary anatomy. The use of intraoperative methylene blue could be a low-cost and simple alternative for ensuring a safe laparoscopic cholecystectomy.
